# Cloning and Expression of *B. mellitensis bp26* Gene in *Lactococcus lactis* as a Food Grade Vaccine

**Published:** 2019

**Authors:** Maryam Azizpour Maghvan, Parvaneh Jafari, Seyed Davood Hoseini, Ali Mohammad Behrozikhah

**Affiliations:** 1. Department of Microbiology, Islamic Azad University, Arak Branch, Arak, Iran; 2. Razi Vaccine and Serum Research Institute, Agricultural Research, Education & Extension Organization, Arak, Iran; 3. Razi Vaccine and Serum Research Institute, Agricultural Research, Education & Extension Organization, Karaj, Iran

**Keywords:** Brucellosis, Exotoxins, *Lactococcus lactis*, Vaccines

## Abstract

**Background:**

Brucellosis is still an important health problem in under developing countries and researches for finding efficient vaccine are going on. *Brucella melitensis (B. mellitensis) bp26* gene is a good candidate for brucellosis vaccine and investigations showed that *Lactococcus lactis* (*L. lactis)* with several positive characteristic are attractive for protein expression as a live delivery vectors. These fast growing bacteria need no aeration, are easy to handle, have no exotoxin, endotoxin and protease, so the cost of culturing is inexpensive.

**Methods:**

*B. mellitensis bp26* gene was cloned in food grade pNZ 8149 vector and expressed in *L. lactis* NZ 3900.

**Results:**

Results showed that we can produce a food-grade recombinant *L. lactis* producing the *B. melitensis* BP26 protein.

**Conclusion:**

In this study, for Future evaluation about ability of *L. lactis* as a live delivery vector, a food-grade recombinant *L. lactis* producing the *B. melitensis* BP26 protein was produced.

## Introduction

Brucellosis is zoonotic diseases which made health and economic problem in many countries 1. In industrialized nations because of routine screening of domestic livestock and animal vaccination brucellosis in humans and livestock are relatively uncommon. Up to now no human vaccines are available, and current animal vaccines are both virulent in humans and lack clinical efficacy 2. Therefore, an efficient, economical and easily managed vaccine needs to be developed.

Researchers revealed that *Brucella* *melitensis (B. melitensis) bp26* gene is a good immunogen and can be candidate for *Brucella* spp vaccine 3. This gene encoding the 28 *kDa* periplasmic protein is named BP26, CP28 or Omp28 and is a target molecule to detect anti-*Brucella* antibodies 4,5. To date, *Lactococcus lactis (L. lactis)* is attractive live delivery vector through mucosal routes for delivering bioactive proteins. *L. lactis* enters through M cells and multiplied within phagocytic cells so releasing and spreading in deeper layer was occurred. Therefore induction of immune responses against *L. lactis* antigens was Getting Started 6–10. PNZ8149 was used as the broad host range vector. This vector produces a cytoplasmic protein and to prevent protein removal by digestive enzymes or by other factors in the digestive tract, this protein was not designed to be secreted or attached to the cell surface of bacteria. Therefore, after entering of this recombinant bacterium through the M cells and up taking *via* phagocytic cells, the probability of induction the immune system, through BP26 protein, is higher 10.

In this study for first time, *B. mellitensis bp26* gene was cloned into the PNZ 8149 vector and expressed in *L. lactis* NZ 3900 for used as a research experimental tool to find a good vaccine candidate.

## Materials and Methods

### Bacterial strains and growth conditions

Any bacterial strains and plasmids used in this study are showed in table 1. All *L. lactis* strains were grown at 30*°C* on M17 media (Merck, Germany) containing 0.5% glucose (M17-glu) or lactose (M17-lac). All *Escherichia coli* (*E. coli)* DH5α strain were grown at 37*°C* on Luria-Bertani (LB) medium (Merck, Germany) containing 50 *μg/ml* Ampicillin or 50 *μg/ml* kanamycin.

**Table 1.** Bacteria strains and plasmids used in this study

**Strains or plasmids**	**Relevant characteristics**	**Source**
**Strains**		
*E. coli*DH5α	Host	Fermentas Kit
*L. lactis*NZ39000	Host	Mo Bi Tec Co
*L. lactis*NZ8149	harboring pNZ8149 plasmid	Mo Bi Tec Co
*E. coli*DH5α	harboring recombinant pET28a +bp26 plasmid	Our lab preserved (3)
**Plasmids**		
pTZ57R/T	*E. coli*TA cloning vector	Fermentas Kit
pNZ8149	Food grade *L. lactis* lacF selection marker,	Mo Bi Tec Co

### Amplification of bp26 gene

To amplify the *bp26* gene, one pair of PCR primers was designed based on sequences published in Gene Bank (accession No. JF918758.1), and the restriction endonuclease sites of XbaI and SphI were added to both ends of the modified bp26 protein gene e based on the structure of PNZ8149 (forward: GCATGCATGA ACACTCGTGC and reverse: TCTAGATTACTTGAT TTCAAAAACGAC). Template DNA (pET28a+bp26) preserved by Our lab 3. The PCR was performed initial denaturation at 95*°C* for 2 *min*, followed by 34 cycles of 95*°C* for 1 *min*, 58*°C* for 1 *min*, and 72*°C* for 1 *min*. with Extra polymerization in 72*°C* for 30 *min*. The PCR product consisting 753 *bp* was checked using agarose gel electrophoresis and then purified using a Fermentas Silica Bead DNA Gel Extraction Kit.

### Cloning and transformation

The PCR product was cloned in to pTZ57R/T vector and transformed in *E. coli* DH5α competent cells. The recombinant pTZ57R/T plasmid was extracted and digested with two restriction enzymes (SphI/NEB Bio lab and XbaI/Fermentas Digestion Enzyme). At the same time the pNZ8149 plasmid was digested with both SphI and XbaI and purified. The purified desire was inserted into the pNZ8149. Competent *L. lactis* NZ39000 cells were then electro-transformed with the recombinant plasmids (Gene e-Pulser; Bio-Rad, Hercules, CA, USA) and cultured on Elliker agarlac bromocresol purple and incubated at 30*°C* for 48 *hr*. Transformants harboring the recombinant plasmids were verified through enzymatic digestion and PCR.

### Expression of recombinant protein

Expression performed according to MoBiTec NICE_ Expression_System and analyzed on 10% SDS-PAGE. To confirm the accuracy of the SDS-Page and protein expression, Western Blot was performed with Nitrocellulose Membrane (Sigma) and using the Trans-Blot SD cell (BIO-RAD). After blocking with TBST (tris-buffered saline, 0.05% Tween-20) buffer containing 5% skimmed milk at 4*°C* overnight, the membranes were incubated with a mouse IgG monoclonal antibody, anti-OMP28, (MyBioSource, Inc, USA) at a dilution 1:500 in phosphate-buffered saline (PBS) at a 37*°C* for 60 *min*. Then, the blots were washed and incubated with 1:2000 dilution of HRP-conjugated rabbit anti-mouse IgG (MyBioSource, Inc, USA) for 60 *min*. Binding was visualized using diaminobenzidine (Merck), according to the manufacturer's instruction.

## Results

### PCR screening

**Figure 1.**
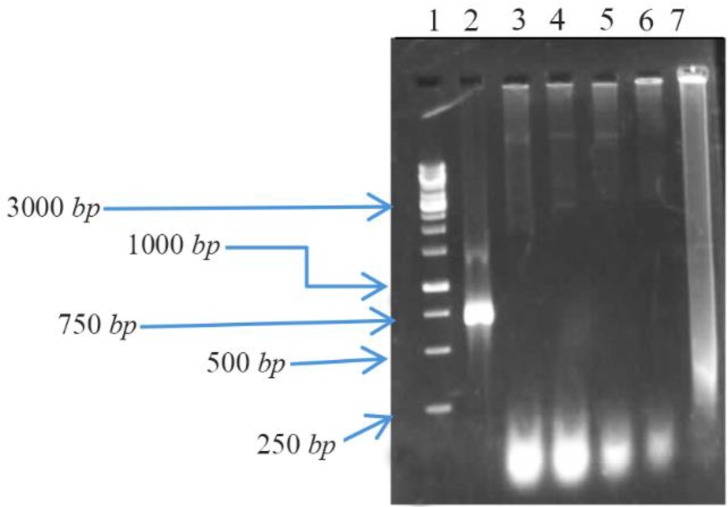
Colony PCR from random selected colonies on 1% agarose gel: Lane 1; Fermentas 1 *Kb* DNA Ladder, Lane 3, 4, 5, 6 and 7; negative colonies, Lane 2; positive colonies.

Results showed that the expected DNA band of the *bp26* gene had been amplified; the PCR product was approximately 753 *bp* in length plus the 12 *bp* restriction sites (Figure 1).

### Digestion screening

**Figure 2.**
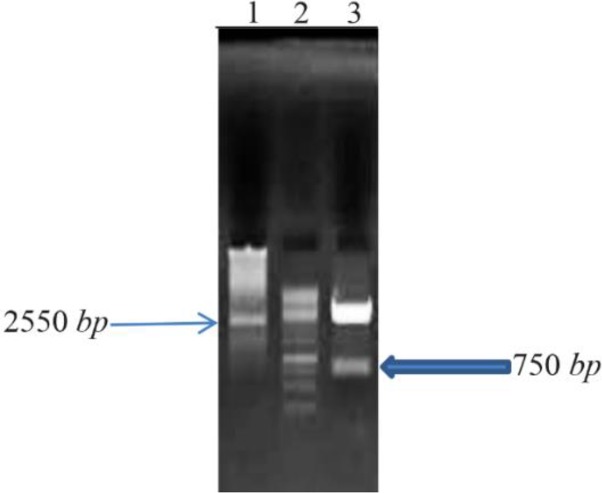
Double-digestion on 1% agarose gel: Lane 1; pNZ 8149, Lane 2; Fermentas 1 *Kb* DNA Ladder, Lane 3; pNZ 8149+ bp26 double-digestion.

Double-digestion confirms the size of *bp26* gene (Figure 2).

### Induced expression of the recombinant L. lactis

**Figure 3.**
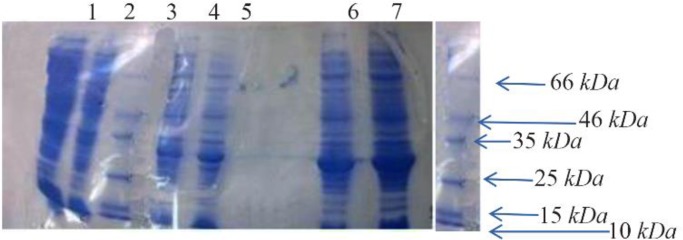
*bp26* gene expression on SDS-PAGE: Lane 1; *L. lactis* with pNZ 8149 vector, Lane 2; *L. lactis* 3900, Lane 3; Fermentas protein Ladder, Lane 4; transformed *L. lactis* with recombinant pNZ 8149+bp26 vector before adding Nisin, Lane 5; transformed *L. lactis* with recombinant pNZ 8149+bp26 vector 1 *hr* after adding Nisin, Lane 6; transformed *L. lactis* with recombinant pNZ 8149+bp26 vector 3 *hr* after adding Nisin, Lane 7; transformed *L. lactis* with recombinant pNZ 8149+bp26 vector 5 *hr* after adding Nisin.

Results of *bp26* gene expression on SDS-PAGE; as it is evident in the figure, with increasing Nisin (1 *ng/ml*) addition time, the amount of protein expression also increases. Protein production increase with Nisin and in 5^th^ *hr* the high level of protein production was seen. Results indicated that the molecular weight of the expressed recombinant protein was approximately 28 *kDa* (Figure 3).

**Figure 4.**
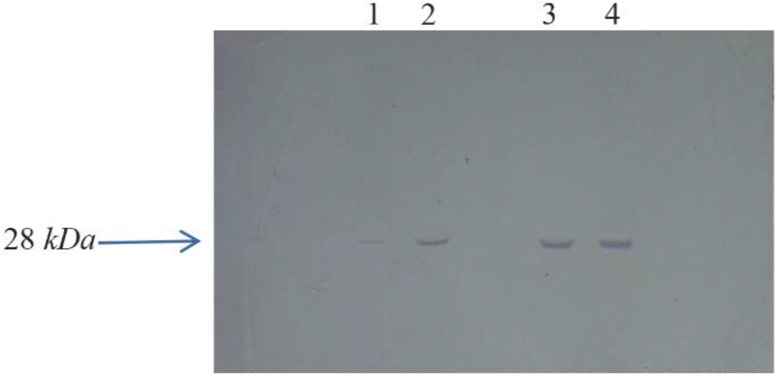
BP26 production was approved by western blot analysis. Lane 1; BP26 before adding nisin, Lane 2; BP26 production 1 *hr* after adding nisin, Lane 3; BP26 production 3 *hr* after adding nisin, Lane 4; BP26 production 5 *hr* after adding nisin.

Western blot results showed that the produced protein was the *B. melitensis* omp28 (Figure 4). The result show that the binding of BP26 protein and its antibody occurred.

## Discussion

Brucellosis is a worldwide zoonotic disease, which remain an important public health concern and causes economic losses in endemic areas 11,12. Vaccination is the most possible way to reduce the transmission in domestic animal herds and humans. The infectious cycles of most pathogenic micro-organisms initiate from mucosal surfaces. So, if colonization and invasion of infectious agents stopped in this stage, the infection does not happen. For this purpose, a vaccine must be made to stimulate mucosal and cellular immunity 13–15. Today, investigations showed that using Lactic Acid Bacteria (LAB) as a live delivery vectors for antigens can induce mucosal immunity and one of the most important candidates to produce mucosal vaccines. In this investigation, we used *L. lactis* 3900 as a gene delivery vehicle. Despite the fact that *L. lactis* is a non-commensal and non-colonizing bacterium at the level of the gastrointestinal tract, it can be easily taken up by M cells, and exhibits adjuvant/immune potentiating activity 16,17. As *Brucella* infections involve mainly bacterial entry through the mucosal routes, the development of successful approaches for oral vaccination could radically alter the current scene of brucellosis 18,19. Most published studies have evaluated the use of live vectors expressing *Brucella* antigens for vaccine delivery at the mucosal gut. At present, several recombinant proteins of *Brucella* have been evaluated as oral vaccine with *L. lactis* and sufficient evidence showed that they can induce protective immunity in mice 19–23. For example, in 2002, Luciana A. Ribeiro *et al* expressed *Brucella abortus* L7/L12 gene in *L. lactis*, under the nisin-inducible promoter 22. In another work, Daniela S. Pontes *et al* in 2003, revealed that a recombinant *Lactococcus lactis* strain producing L7/L12 under the control of nisin inducible promoter when orally administered to BALB/c mice, they could induced local humoral immune response and detected significant levels of anti-L7/L12 specific IgA in feces 21.

In 2012 DarwinSáez *et al*, transformed *Brucella abortus* (*B. abortus)* Cu-Zn Superoxide dismutase (SOD) in *L. lactis* revealed that orally vaccinated mice protected against challenge with the virulent *B. abortus* 2308 strain 19.

## Conclusion

According to the investigations which mentioned above and considering that *B. melitensis* BP26 is a good immunogenic protein 24, in this study, we successfully constructed a food-grade recombinant *L. lactis* producing the *B. melitensis* BP26 protein for future researches about induction of immune response by this protein.
